# A framework for semantics-based situational awareness during mobile robot deployments

**DOI:** 10.3389/frobt.2025.1694123

**Published:** 2025-11-19

**Authors:** Tianshu Ruan, Aniketh Ramesh, Hao Wang, Alix Johnstone-Morfoisse, Gokcenur Altindal, Paul Norman, Grigoris Nikolaou, Rustam Stolkin, Manolis Chiou

**Affiliations:** 1 Extreme Robotics Lab (ERL) and National Center for Nuclear Robotics (NCNR), University of Birmingham, Birmingham, United Kingdom; 2 Birmingham Centre for Nuclear Education & Research (BCNER), University of Birmingham, Birmingham, United Kingdom; 3 Department of Industrial Design and Production Engineering, University of West Attica, Athens, Greece; 4 School of Electronic Engineering and Computer Science, Queen Mary University of London, London, United Kingdom

**Keywords:** situational awareness, semantics, semantic understanding, human–robot teaming, disaster-response robotics, search and rescue robotics

## Abstract

Deployment of robots into hazardous environments typically involves a “human–robot teaming” (HRT) paradigm, in which a human supervisor interacts with a remotely operating robot inside the hazardous zone. Situational awareness (SA) is vital for enabling HRT, to support navigation, planning, and decision-making. In this paper, we explore issues of higher-level “semantic” information and understanding in SA. In semi-autonomous or variable-autonomy paradigms, different types of semantic information may be important, in different ways, for both the human operator and an autonomous agent controlling the robot. We propose a generalizable framework for acquiring and combining multiple modalities of semantic-level SA during remote deployments of mobile robots. We demonstrate the framework with an example application of search and rescue (SAR) in disaster-response robotics. We propose a set of “environment semantic indicators” that can reflect a variety of different types of semantic information, such as indicators of risk or signs of human activity (SHA), as the robot encounters different scenes. Based on these indicators, we propose a metric to describe the overall situation of the environment, called “Situational Semantic Richness” (SSR). This metric combines multiple semantic indicators to summarize the overall situation. The SSR indicates whether an information-rich, complex situation has been encountered, which may require advanced reasoning by robots and humans and, hence, the attention of the expert human operator. The framework is tested on a Jackal robot in a mock-up disaster-response environment. Experimental results demonstrate that the proposed semantic indicators are sensitive to changes in different modalities of semantic information in different scenes, and the SSR metric reflects the overall semantic changes in the situations encountered.

## 1 Introduction

Situational awareness (SA) is vital for robots deployed in the field to function with sufficient autonomy, resiliency, and robustness. This is especially true for human–robot teams (HRTs) in safety-critical applications such as disaster response, remote inspection in unstructured environments, or nuclear operations (Chiou et al., 2022; Ruan et al., 2022; Stolkin et al., 2023). In all cases, humans and robots require SA to make plans or decisions in the context of HRT (e.g., identifying a proper timing to switch control between human operators and robots). Hence, humans and robots need to know and share what is happening in the environment to plan and act in a safe and coordinated manner. Humans and robots (to avoid verbosity, we sometimes use the term “robot” synonymously with the AI or autonomous agents controlling the robot) have distinct strengths and weaknesses in terms of perception, sensory data interpretation, and decision planning and execution in response to those data in real time.

Building on low-level signals from multiple modalities of on-board cameras and sensors, higher-level “semantic” understanding (Ruan et al., 2022) of scenes, environments, and situations must be developed. Often, this higher-level semantic knowledge will be critical for determining subsequent decisions and actions. Recent advances, especially from the computer vision community (Long et al., 2015; Li et al., 2017), have begun to provide autonomous agents with some elements of semantic-level perception. Meanwhile, in real-world robotic systems at present, the intelligence of human operators may often be necessary to correctly interpret and act upon semantically rich situations. In this paper, we propose a framework for robots to acquire semantically enhanced SA that combines with human understanding in an explainable and intuitive way.

**FIGURE 1:**
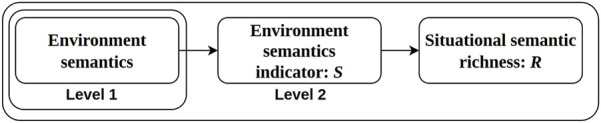
Mobile robot semantic SA.

Human factors SA can be modeled in terms of three levels of awareness (Endsley, 2017): level 1) perception of elements in the current situation, level 2) comprehension of the current situation, and level 3) projection of future status. In the robotics and AI research literature, it is common to use terms such as sensing, perception, scene understanding, semantics, and context (Bavle et al., 2023) instead of SA. There are connections among these related concepts; for example, the concept of perception “elements” in SA can be linked to the “semantics” concept in AI. Hence, although the conventional SA model is designed to represent the awareness of human operators, the SA of an autonomous or semi-autonomous robot can be structured similarly in the scope of semantics. Elements of level-1 SA can be objects, sensor readings, and other low-level semantics (Ruan et al., 2022). The comprehension of the current situation at level 2 corresponds to high-level semantics (Ruan et al., 2022) (see Figure 1). Prediction, planning, or decision-making based on these constitute the main focus of level 3.

Our work aims to build a systematic framework and concepts the following: a) make SA sharing from robot to human easier, practical, and intuitive and b) facilitate the use of semantically enhanced SA in HRT planning and decision-making frameworks. We build upon our previous work that proposed a taxonomy of semantic information (Ruan et al., 2022) and definitions of low-level semantics, high-level semantics, and the context in robot-assisted disaster response.

In this paper, we explore level-2 SA and propose the concept of an “environment semantic indicator” along with an example realization. Each indicator captures the understanding of an environment’s semantics intensity, such as signs of human activity (SHA). Furthermore, we build upon those indicators to develop an aggregated metric, the “situational semantics richness” (SSR), which expresses the overall intensity and plethora of semantic information in an environment.

## 2 Related work

Early studies in human factors analyzed SA based on human feedback after trials (Stanton et al., 2017), such as by using the Situation Awareness Global Assessment Technique (SAGAT) (Endsley, 1988) or the Situational Awareness Rating Technique (SART) (Taylor, 2017). Endsley and Mica R. proposed a three-level SA model, which is widely accepted (Endsley, 1995). SA is a subjective concept based on objective reflections of the environment, which means that everyone may understand the situation differently. Thus, building a generalized framework to regulate understanding is essential. A subjective scoring or weighting system that delivers a subjective understanding is commonly used to differentiate each situation. For instance, Hooey et al. (2011) built a heuristic scoring system and gave weights to different “situational elements” to model the SA obtained from aircraft pilots. McAree et al. (2018) gave examples of formalizing some specific awareness, such as the position and air environment (consisting of air traffic, airspace restrictions, and weather), using a scoring system.

These works show how humans obtain SA. Elements of such approaches can be generalized to robot SA. Some research workers discuss combined human SA and robot SA or view the problem from a global perspective in the context of human–robot interaction (HRI) (Dini et al., 2017). Other research workers use ontology to obtain the SA. Ontology concerns what kinds of things exist, how they can be organized, and what relationships exist between them (Huang et al., 2019; Tenorth and Beetz, 2017). Armand et al. (2014) modeled simple situations on the road and crossroads using ontology. Authors categorize road contexts into “mobile entities,” “static entities,” and “context parameters” that describe the relationship between entities from the spatiotemporal scope. Rules are established for the vehicle when the combination of road contexts changes. Ontologies are intuitively straightforward for modeling situations and are easy to understand. However, the ontology models are built on simplified or specific situations. They may have problems in complex environments and unexpected situations. Hence, robots need multiple inference methods to obtain SA (Tenorth and Beetz, 2017). Alternatively, probabilistic methods can be used to model the environment and generate SA (Shuang et al., 2014). Nguyen et al. (2019) compared multiple SA measurements that formalize the SA. Apart from a human perspective, the authors also review the SA for unmanned aerial vehicles (UAVs). They claim that most SA studies focus on the human perspective and indicate that there are limited methods to frame and obtain UAV SA. Senaratne et al. (2025) systematically discussed the dynamic nature of team SA and the factors that affect SA. Meanwhile, our work focuses on using high-level semantics to facilitate SA in the scope of HRT.

In general, most of these SA assessments define metrics highlighting the flexibility and the importance of expert knowledge. However, there are limited works on how robots perceive high-level semantics and how robots can aggregate those semantics into coherent and usable metrics reflecting the overall SA and context. Unlike human SA research, most robot SA research still focuses on addressing specific problems from one specific scope, such as electromagnetic jamming security (Gao et al., 2020) or failure conditions (Ginesi et al., 2020; Ghezala et al., 2014). In contrast, we propose a general framework and an example realization for an aggregated metric of SA, which enables robots to understand the overall environmental situation and can be generalized to different deployment tasks.

## 3 Problem formulation and concept definition

Here, we assume that the deployed robots need to perform tasks such as scanning a damaged building (Kruijff et al., 2012), surveying and sampling contamination in a hazardous site (Nagatani et al., 2013), remotely inspecting and monitoring facilities, or searching for human victims (Murphy, 2014; Ruan et al., 2022) in the context of disaster response. Robots can be tele-operated (Chiou et al., 2022), semi-autonomous [e.g., variable autonomy (Reinmund et al., 2024; Methnani et al., 2024), mixed-initiative (Chiou et al., 2021), or shared control (Pappas et al., 2020) paradigms], or run fully autonomously. In all cases, robots need SA to make plans or decisions D in the context of HRT. Hence, there is a mapping I:S↦D between a set of environment semantics S and the decisions D .

Specifically, $S=\left\{S_{1},S_{2},\ldots ,S_{i},\ldots S_{n}\right\},\left(n>=1,0<S_{i}<1\right)$ comprises a set of different possible types of environment semantics, such as SHA, noise for LiDAR, or detection of hazards, where n denotes the number of semantic indicators in S . Note that S can be configured to contain many different kinds of semantic information, as may be appropriate to different types of robot missions and application domains. Without the loss of generality, in this paper, we use the example of disaster response to provide an intuitive illustration of how this framework can be applied in a practical task. As examples of possible S , we present experiments in which we use the following: $S_{1}=S\left(noise\right)$ , $S_{2}=S\left(risk\right)$ , $S_{3}=S\left(SHA\right)$ , and $S_{4}=S\left(radiation\right)$ . These are examples of environment semantics that can be useful in disaster response and remote inspection missions (Ruan et al., 2022).

A significant challenge is that it is nontrivial to parameterize a framework for a mapping I , which can directly map semantics S onto decisions D . Therefore, the key idea of this paper is to introduce an intermediary term, which we call SSR R . The term R serves to aggregate the environment semantics combinations in S , which can then assist with bridging toward the decision set D . We define the function $R=f\left(S_{i},W_{i}\right)$ , where $W_{i}$ is a set of weights that reflects the relative impact of each type of semantic information. Note that this paper focuses on addressing the problem of progressing from S to the intermediary term R . The next challenge of formulating a relationship between R and D will form the subject of a future paper and is out of the scope of the present paper. However, in this paper, we show how the formulation of S , and its mapping to the intermediary term R , is already a useful tool in its own right for assisting SA in HRT missions.

## 4 Environment semantic indicator

### 4.1 Laser noise intensity

Many unmanned ground vehicles (UGVs) rely on lasers for autonomous navigation. However, laser noise potentially affects the navigation. We adapt the method to obtain laser noise variance $\left(\sigma_{\mathrm{noise}}^{2}\right)$ in our previous work (Ramesh et al., 2022). It is calculated by convolving the laser map image with a 3 × 3 mask and applying summations on the resultant matrix. Then, we adapt the noise variance into a sigmoid function (see Equation 1 and Ramesh et al., 2022) to obtain the laser noise intensity S(noise) . To give a rough indication of the scale, when operating our mobile robot in our laboratory’s mock-up disaster scene testing environment, we notice that a $\sigma_{\mathrm{noise}}^{2}>=1.4$ is sufficient to severely disrupt autonomous navigation, causing the robot to stop. We use this critical value to help inform choices for parameters a and b to tune the system to our robot and testing environment. Combining the preliminary test results, the laser noise intensity is designed as follows:

$$\begin{array}{c} S\left(noise\right)=\frac{1}{1+\exp\left(-a\cdot \sigma_{\mathrm{noise}}^{2}+a\cdot b\right)}, \end{array}$$ (1)

where a=5 and b=1 , to obtain a curve that responds to medium inputs but is not oversensitive to low or high inputs.

### 4.2 Risk to robots

Risk to robots can be quantified based on the hazard level and hazard length (Soltani and Fernando, 2004). The hazard level refers to how dangerous the hazard is to the robot, and the hazard distance refers to the distance to the object. Furthermore, time may also affect the risk level, such as the accumulated dose received from the radiation sources.

In this work, we assume that the robot can detect potential risks by detecting hazmat signs that commonly exist in hazardous environments. In general, humans and robots face similar risks. However, considering the slight difference between the risk to humans and risk to robots, the categorization might differ but can be trivially adapted to reflect human risks, expert knowledge (e.g., by first responders), or different scenarios. We categorize the hazmat signs into three levels heuristically: low risk or no risk for the robot (e.g., poison, infectious substance, nonflammable gas, and inhalation hazard), medium risk (delayed hazard to a robot or they can be high risks under certain occasions, e.g., corrosive, radioactive, dangerous when wet, oxygen, and organic peroxide), and high risk (immediate hazard to the robot, e.g., explosives, flammable solid, flammable gas, and spontaneously combustible material). Other risks that are not included can also be added trivially in different scenarios.

Intuitively, distance is a factor that relates to the risk intensity. Referring to the relationship between the radiation strength and distance (Voudoukis and Oikonomidis, 2017), we apply a similar model risk to robots S(risk) by using the inverse square law (see Equation 2):

$$S\left(risk\right)=\overset{n}{\underset{i=1}{\sum }}\frac{H_{j}}{d_{i}^{2}},$$ (2)

where n is the number of detected signs, i is the label of each detected sign, d refers to the distance to the robot, j refers to the level of the corresponding signs, and $H_{j}$ refers to the risk intensity of each level of hazmat sign.

Then, we normalize the risk score by applying the sigmoid function. The reason for using the sigmoid function is when the x-axis closes to infinity, the slope of risk is low and accords with human common understanding; that is, the environment that has six high-risk objects has a similar S(risk) as the environment containing five high-risk objects. The normalized score is as follows (see Equation 3):

$$S{\left(risk\right)}_{\mathrm{norm}}=\frac{1}{1+e^{-aS\left(risk\right)-b}},$$ (3)

where a and b are used for tuning the functions. In our experiments, we set them as a=0.09 and b=−4.8 heuristically to get a meaningful and usable curve. Experts can tune a and b for different tasks.

### 4.3 Signs of human activity

Robots might not always directly detect human victims in the environment (e.g., trapped under debris or occluded by objects). Hence, robots must identify clues to find victims. SHA are considered a potential factor in finding people (Yang et al., 2018). We use human belongings, such as mobile phones, keys, and watches, as indicators of human activity. Intuitively, the dispersion of personal belongings makes a difference to the SHA. Thus, the SHA model is developed from two aspects: the class of objects and the dispersion of the objects.

We design three classes of objects to differentiate the impact of different human belongings: i) high impact means there is a high chance for these objects to be found on the human body (e.g., glasses, key, cell phone, and watch); ii) medium impact means there are chances for these objects to be found in proximity to the human body (e.g., cap, mask, and wallet); iii) low impact means there is a high chance for these objects not be carried on the human body (e.g., laptop and backpack). This heuristic classification is an example of realization for our framework, and experts can adjust it.

Based on the above, we propose the following model to estimate the SHA score S(SHA) (see Equation 4):

$$\begin{array}{c} dispersion=\overset{n}{\underset{i=1}{\sum }}|d_{i}-d_{\mathrm{average}}|,\\ S\left(SHA\right)=\overset{n}{\underset{i=1}{\sum }}\frac{P_{j}}{dispersion}, \end{array}$$ (4)

where n is the number of detected objects, i is the label for each object, $d_{i}$ is the distance from the corresponding objects to the robot, $d_{\mathrm{average}}$ is the average distance of all the objects, j is the label of class for objects, and $P_{j}$ refers to the impact of corresponding objects.

Similarly, we normalize the S(SHA) using the sigmoid function (see Equation 5):

$$S{\left(SHA\right)}_{\mathrm{norm}}=\frac{1}{1+e^{-aS\left(SHA\right)-b}},$$ (5)

where a and b are used for tuning the function. In our experiments, we used a=10 and b=−0.5 to obtain a curve that has similar sensitivity characteristics as Equation 1. The parameters can be adjusted when expert knowledge is involved.

### 4.4 Radiation

Not only humans but robots may also be affected by radiation (Nagatani et al., 2013). The highest risk will be to the onboard electronics, as radiation can cause disruptions, malfunctions, or even complete failure of electronic components. These issues can be addressed by applying appropriate shielding and mitigation techniques or designing radiation-hardened robots. However, radiation shielding is typically extremely heavy, leading to large and bulky robots that may be impractical in certain tasks, such as entering a hazardous zone with a small robot via a small door or aperture. Alternatively, we might monitor the radiation strength in deployment so that robots and humans can avoid exposure.

The risk associated with radiation depends on the distance from the source and the radiation’s type, strength, and energy. It is important to distinguish between the dose rate and the total integrated dose. The dose rate is commonly measured in sievert per hour (Sv/h) and microsievert per hour (μSv/h) , which is the rate at which the radiation is received at a given moment. The total integrated dose measured in sieverts results from the accumulation of radiation over time.

We designed a mapping I:G↦S(radiation) . It translates the raw readings that require radiation backgrounds for understanding to a matric ranging from 0 to 1. G is a set of gamma radiation dose rates G in μSv/h from the sensor, and S(radiation) denotes the radiation strength. Specifically, the sigmoid function is applied in the mapping I to normalize the S(radiation) (see Equation 6):

$$S{\left(radiation\right)}_{\mathrm{norm}}=\frac{1}{1+e^{-aG-b}},$$ (6)

where a=8 and b=−8 . Specifically, S(radiation)=0 refers to no radiation, and S(radiation)=1 refers to radiation that can instantly damage the robot. This tuning setting takes into account the effects of background radiation (usually under 0.4 μSv/h ) and decreases the impact of it.

## 5 Situational semantics richness

**FIGURE 2:**
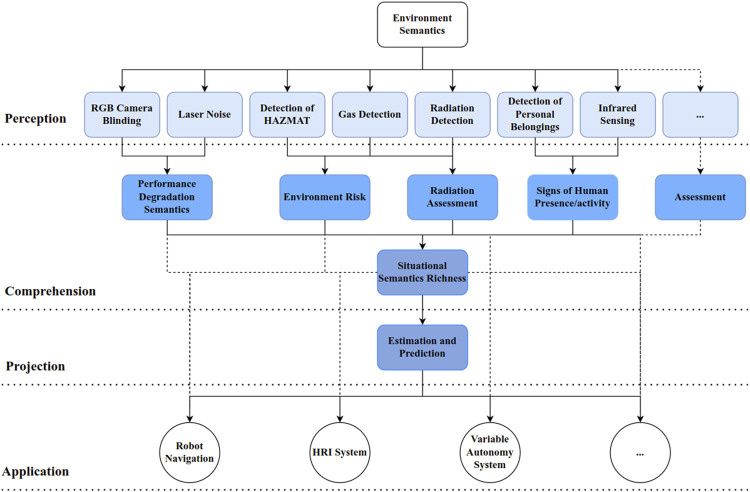
Semantics-based SA framework: the light blue box refers to low-level semantics, the cornflower blue box refers to high-level semantics, and the dark blue box refers to context. Black dashed lines indicate the potential connections among different levels.

With the semantics in Section 4, we propose a framework (shown in Figure 2) that fuses the semantics of the environment at a higher level, that is, a metric that describes the overall status of the environment in an aggregate representation.

In real-world situations, if we do not have a dataset, applying data-driven approaches is impractical or intractable. To the best of our knowledge, no dataset involves all the environment semantics; that is, it is not feasible to build a parametric model and train an end-to-end network to assess the situation. Moreover, SA is a subjective understanding and needs to be intuitive and explainable, especially in safety-critical and hazardous applications. Thus, we must capture human understanding into our framework. It is common practice to build a heuristic-based system that comprises important factors and expert knowledge to obtain SA. It is straightforward to adjust. The SSR is proposed based on this idea. We obtain the score of each environment semantic indicator and adapt it into the proposed framework by developing the SSR score, which expresses the overall intensity and plethora of semantic information from the environment.

We obtain a set of normalized metrics S from 0 to 1 in Section 4. In different scenarios, different semantics might have different importance. To address this, we assign an importance weight $W_{i}$ for each $S_{i}$ . Tuning $W_{i}$ provides the framework extensibility to different deployment cases and tasks while enabling leveraging expert knowledge. In our experiment, we adopt the exponential weight $W_{i}=e^{\frac{S_{i}}{0.99}}$ to emphasize the environment semantics with a higher score, where $S_{i}$ is the score of each environment semantic and i is the label of environment semantics. The exponential weight lets high-score semantics have a higher impact in the final SSR score. Then, we define the situational semantics richness R as follows (see Equation 7):

$$\begin{array}{c} R=\overset{n}{\underset{i=1}{\sum }}W_{i}\times S_{i}, \end{array}$$ (7)

where n denotes the number of environment semantics. We normalize the R using a sigmoid function to obtain $R_{\mathrm{norm}}$ (see Equation 8), which can enable a better understanding of the SSR intensity by humans:

$$\begin{array}{c} R_{\mathrm{norm}}=\frac{1}{1+e^{-aR-b}}, \end{array}$$ (8)

where a and b can be set as 10 and -0.5, correspondingly, to fit the range (0,1).

To address the effects of unreliable scores caused by noise or false detection, we process the normalized SSR score by involving historical data. We apply an attention mechanism regression to comprise the past SSR scores and emphasize the impact of the latest score. The attention mechanism was first proposed by Èlizbar A. Nadaraya (Nadaraya, 1964) and Geoffrey S. Watson (Watson, 1964), and it has been widely used for nonparametric estimation and deep learning (Vaswani et al., 2017). It runs like the human’s attention to indicate which value or factor deserves more focus among the rest of the data.

In our case, we can obtain a set of R in time sequence $R_{t}=\left\{R_{1},R_{2},\ldots ,R_{t}\right\},t>0$ , where t refers to the current timestamp. Then, the estimated situational semantics richness $\hat{R_{t}}$ at time t can be defined as follows (see Equation 9):

$$\hat{R_{t}}=\overset{n}{\underset{i=1}{\sum }}\frac{K\left(t-t_{i}\right)}{\sum_{j=1}^{n}K\left(t-t_{j}\right)}\times R_{t},$$ (9)

where K is the kernel function and $t_{i}$ is the timestamp of different $R_{t}$ . Hence, $t-t_{i}$ refers to their individual time gap to the current time. If we apply the Gaussian kernel, which is mostly used in Equation 10, the estimated situational semantics richness $\hat{R_{t}}$ is (see Equation 10):

$$\begin{array}{c} K\left(u\right)=\frac{1}{\sqrt{2\pi }}\exp\left(-\frac{u^{2}}{2}\right),\\ \hat{R_{t}}=\overset{n}{\underset{i=1}{\sum }}softmax\left(-\frac{1}{2}{\left(t-t_{i}\right)}^{2}\right)\times R_{t}, \end{array}$$ (10)

where u refers to $t-t_{i}$ in this case.

In the experiment, we selected the time window of five latest $R_{t}$ , which means n=5 . According to the features of the applied attention mechanism, the older the sample is, the less impact it has on the final score. Hence, the five latest samples are enough to refer. Considering the sampling rate limitation from the radiation sensor (1 Hz), the updating rate of the SSR score is synchronized to 1 Hz. Hence, the tuned situational semantic richness $\hat{R_{t}}$ refers to the $R_{t}$ in 5 s, and we apply the $\hat{R_{t}}$ as the final SSR score. The time complexity of the whole process is O(n) , and the space complexity is O(n) as well, which means that it is an efficient algorithm in the scope of computation.

## 6 Experiments

We tested our framework intending to evaluate the following: i) if the framework can accurately perceive each semantics and their changes separately and ii) if the framework is robust and can adapt in an environment with multiple levels and types of semantic indicators.

We used a Jackal mobile robot with an Intel I5 CPU and GTX 1650TI GPU onboard. The framework is built based on the ROS Noetic system. We ran the framework directly on the Jackal to avoid image transferring to the offsite computer. Additionally, sensors, including a real-sense D435i camera, Velodyne vlp-16 Lidar, and Hamamatsu Gamma Sensor C12137, were mounted on the Jackal. We applied the Yolact (Bolya et al., 2019) as our vision model, providing object detection and instance segmentation results. We made some modifications to the system to enable us to attach depth data to each detected object by aligning the RGB image and depth image. The Hamamatsu Gamma Sensor C12137 is specifically designed to measure gamma radiation in the range 0.03 MeV–2 MeV and dose rate up to 100 μSv/h . Even though the robot would be able to detect high-strength radiation sources from a distance, constrained by regulations from the university, sadly, we had to use a low-strength radiation source (uranium rock) that cannot be detected from long distances (over 10 cm). Stronger sources are not available in our project.

We assume that the robot has a prior map from the SLAM, but SLAM is outside the scope of this paper. The proposed environment semantics were placed in the environment after the mapping. Then, we predefined a set of waypoints that the robot has to navigate. Autonomous navigation was applied because we would like to keep similar trajectories of the robot in corresponding trials.

### 6.1 Experiment I

#### 6.1.1 Implementation

**FIGURE 3:**
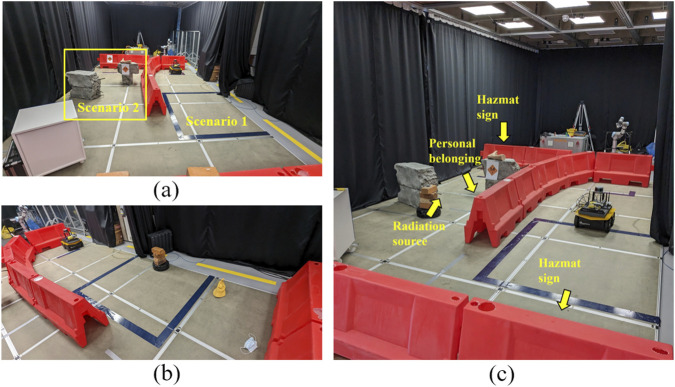
**(a)** Layout for experiment I. The dark blue box area (scenario 1) on the ground is used for laser noise or the radiation source (uranium rock). The yellow box area (scenario 2) is used for hazmat signs or personal belongings. **(b)** Layout for two environment semantics scenarios of experiment II. **(c)** Layout for three environment semantics scenarios of experiment II. In the picture, some environment semantics are covered by red barriers.

We tested the framework in the scenarios with single environment semantics in experiment I. We set two scenarios separately in the area (see Figure 3a). In each scenario, only one environment semantics was added; that is, each environment semantics is independent, and only one semantics with a big impact can be perceived at any time.

Specifically, we defined three cases to differentiate the intensity of the environment semantics: low (radiation and noise), medium (radiation and SHA), and high (risk and noise), which correspond to the levels of environment semantics. For instance, the high case refers to risk and noise that can be detected at a high level and the medium case refers to radiation and SHA detected at the medium level. Due to the nature of the uranium rock, the high-strength radiation scenario needed teleoperation and positioning of the robot close to the source to simulate the situations in which the sensor receives a high dose rate. In addition, we applied longer distances to simulate medium and low cases. We ran the robots 10 times in each of the three cases.

### 6.1.2 Results

**FIGURE 4:**
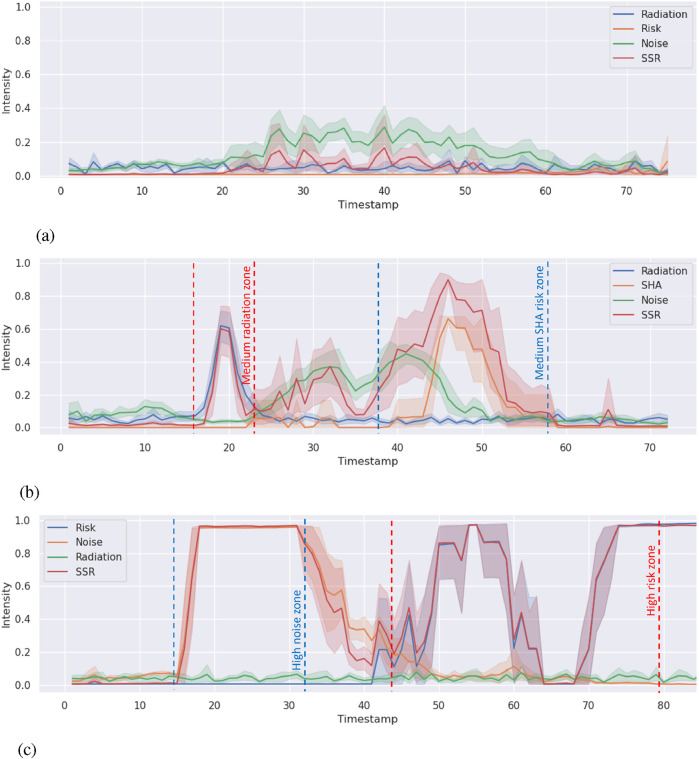
SSR and environment semantics intensity timeline. The lines refer to the average SSR environment semantics intensity in five runs. The shade areas show the minimum and the maximum range. Zones between the dashed lines refer to the corresponding semantics detected from the environment. **(a)** SSR and environment semantics (radiation and noise) intensity timeline in LOW case. **(b)** SSR and environment semantics (radiation and SHA) intensity timeline in MEDIUM case. **(c)** SSR and environment semantics (risk and noise) intensity timeline in HIGH case.

We collected data, including the scores of all environment semantic indicators and timestamps. To examine if the framework can differentiate environments with different levels of semantics, we generated the timeline of the SSR score and environment semantics in Figure 4. Note that in Figure 4c, there is a deep flat between 60 and 70 s. It was caused when the robot turns momentarily and faces the black curtain on the left. At that point, the camera, which is constrained by the view of the field, was unable to see the last hazmat sign on the red barrier until it moved forward and faced the sign. To the best of our knowledge, there is no prior baseline or dataset to compare with. The figures from the different cases reveal that the framework is capable of correctly outputting the corresponding SSR scores in the individual environment semantics scenario. When the robot reached the scenario, the environment semantics indicator responded in time, and the SSR score was affected more by the semantics with higher scores, as designed; that is, the SSR score tracked the environment semantics with the highest score and magnitude. When zooming in specific environment semantics zones, we notice that low-intensity semantics do not affect the SSR score much if medium- or high-intensity semantics exist. We apply variance-based sensitivity analysis (Sobol, 2001) in the medium and high cases (see Figures 4b, c). Specifically, we calculate the global sensitivity (first-order index S1 and total-effect index $S_{t}$ ) of each environment semantics indicator to the SSR score in Table 1. We skip the low case to save space, as the low case is not that important in real deployments as long as no significant error has been found. The analysis showed similar S1 and $S_{t}$ results. The individual semantics of the corresponding zones show strong sensitivity (over 0.7) to the SSR score. It indicates that the SSR score can respond quickly and accurately to single semantics changes in the environment. The medium- or high-intensity semantics dominate the impact and lead the changes in the SSR score. Our framework was tested to capture the changes in individual semantics and reflect correctly on the SSR score.

**TABLE 1:** Sensitivity analysis ( S1 and St ) of environment semantics with the SSR score in experiment I.

Case	Semantic indicator	S1	St
Medium	Radiation (red zone)	0.997	0.997
	SHA (blue zone)	0.977	0.978
High	Noise (blue zone)	0.997	0.997
	Risk (red zone)	0.766	0.769

### 6.2 Experiment II

#### 6.2.1 Implementation

We tested the framework’s performance in scenarios with concurrent multi-environment semantics in experiment II. We designed 12 scenarios in this experiment (see Table 2). Specifically, we select all the possible two-semantics combinations with high- and medium-level intensity and two three-semantics combinations. The scenarios covered a wide spectrum of situations that robots may encounter. We did not test with all of the semantics concurrently as we were constrained by our vision system (train the hazmat and personal belonging detection separately). Additionally, the noise generation design (Ramesh et al., 2023) has only two levels of intensity available. High refers to deliberately adding laser noise into the scenario. Medium refers to no artificial laser noise being added (e.g., normal noise caused by turning the robot). Low refers to background noise. In the scenarios with two environment semantics, we used the corresponding area in Figure 3b. The dark blue box on the ground was used for laser noise or the radiation source. Hazmat signs were put on the right red barrier, and personal belongings were scattered in front of the right red barrier. In the scenarios with three environment semantics, we applied laser noise in the dark blue box, hazmat signs, and radiation source, as shown in Figure 3c and personal belongings were scattered around the gray bricks. The robot ran five times in each scenario.

**TABLE 2:** Combinations of environment semantics in different scenarios in experiment II.

Scenario	Radiation	Risk	SHA	Noise
1	High	—	—	High
2	Medium	—	—	Medium
3	High	High	—	—
4	Medium	Medium	—	—
5	—	High	—	High
6	—	Medium	—	Medium
7	—	—	High	High
8	—	—	Medium	Medium
9	High	—	High	—
10	Medium	—	Medium	—
11	High	—	High	High
12	Medium	Medium	—	Medium

### 6.2.2 Results

**FIGURE 5:**
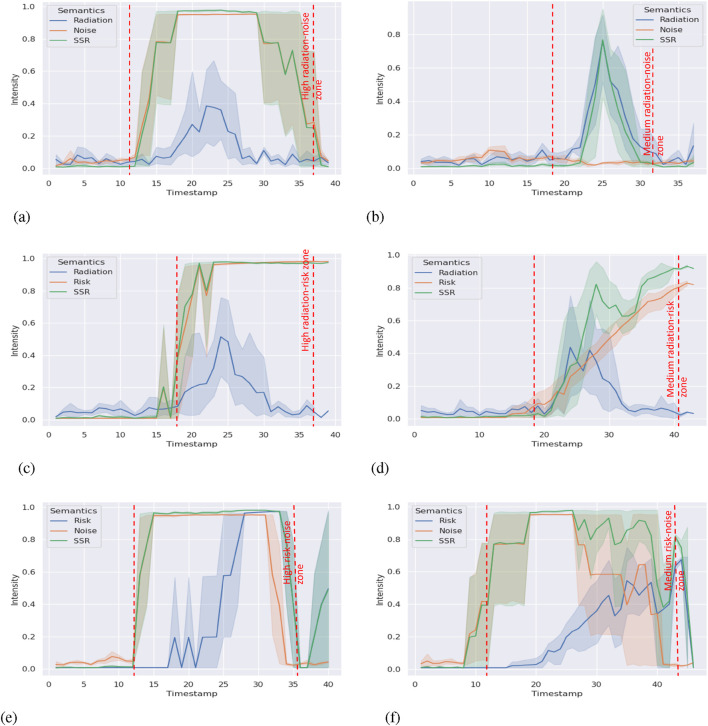
SSR and environment semantics intensity timeline (part 1). **(a)** Radiation–noise combination with high intensity(Scenario 1). **(b)** Radiation–noise combination with medium intensity(Scenario 2). **(c)** Radiation–risk combination with high intensity(Scenario 3). **(d)** Radiation–risk combination with medium intensity(Scenario 4). **(e)** Risk-noise combination with high intensity (Scenario 5). (f) Risk-noise combination with medium intensity(Scenario 6).

**FIGURE 6:**
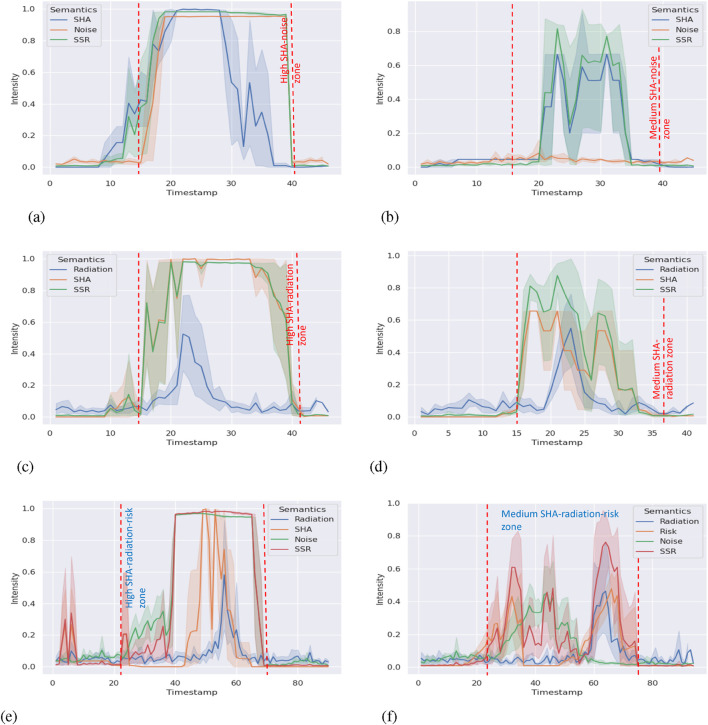
SSR and environment semantics intensity timeline (part 2). **(a)** SHA–noise combination with high intensity (Scenario 7). **(b)** SHA–noise combination with medium intensity(Scenario 8). **(c)** SHA–radiation combination with high intensity(Scenario 9). **(d)** SHA–radiation combination with medium intensity(Scenario 10). **(e)** SHA–radiation-noise combination with high intensity(Scenario 11). **(f)** Risk–radiation-noise combination with mediumintensity (Scenario 12).

We collected data on all environment semantic indicators, SSR scores, and timestamps in experiment II. We aligned the data in each scenario with the timestamp to reduce the error caused by mismatching. The processed results are shown in Figures 5a–f, 6a–f. The shade zone reveals the range of semantic indicators and the SSR score. The solid lines refer to the mean from five trials.

Because of the limitation of the sensitivity analysis (unable to reflect the sensitivity in the complex system with multiple factors), we analyzed individual environment semantics correlations to the SSR score. Our framework is nonlinear. Hence, we calculate Spearman’s rank correlation coefficient (ρ) with corresponding bootstrap 95% CI in Table 3 and Kendall’s rank correlation coefficient (τ) with corresponding bootstrap 95% CI in Table 4 to examine the relationship among them. All the p-values are much lower than 0.05. So, we did not list them in the table.

**TABLE 3:** Spearman’s rank correlation coefficient (ρ) of environment semantics with the SSR score and bootstrap 95% CI in experiment II.

Scenario	Radiation	Risk	SHA	Noise
1	0.75 [0.65, 0.82]	—	—	0.81 [0.74, 0.85]
2	0.86 [0.80, 0.91]	—	—	0.22 [0.07, 0.37]
3	0.67 [0.57, 0.75]	0.80 [0.77, 0.82]	—	—
4	0.56 [0.45, 0.67]	0.88 [0.85, 0.90]	—	—
5	—	0.73 [0.68, 0.75]	—	0.67 [0.56, 0.75]
6	—	0.53 [0.41, 0.62]	—	0.76 [0.72, 0.79]
7	—	—	0.83 [0.77, 0.86]	0.79 [0.75, 0.82]
8	—	—	0.78 [0.71, 0.84]	0.46 [0.35, 0.56]
9	0.68 [0.58, 0.76]	—	0.87 [0.83, 0.89]	—
10	0.67 [0.56, 0.75]	—	0.79 [0.74, 0.84]	—
11	0.53 [0.47, 0.60]	—	0.44 [0.36, 0.54]	0.91 [0.88, 0.92]
12	0.42 [0.34, 0.50]	0.63 [0.57, 0.68]	—	0.50 [0.40, 0.53]

**TABLE 4:** Kendall’s rank correlation coefficient (τ) of environment semantics with the SSR score and bootstrap 95% CI in experiment II.

Scenario	Radiation	Risk	SHA	Noise
1	0.62 [0.54, 0.69]	—	—	0.60 [0.53, 0.65]
2	0.70 [0.64, 0.76]	—	—	0.18 [0.08, 0.28]
3	0.52 [0.45, 0.60]	0.57 [0.53, 0.61]	—	—
4	0.43 [0.34, 0.51]	0.73 [0.69, 0.77]	—	—
5	—	0.53 [0.50, 0.55]	—	0.51 [0.43, 0.58]
6	—	0.42 [0.36, 0.52]	—	0.52 [0.48, 0.57]
7	—	—	0.67 [0.62, 0.71]	0.57 [0.52, 0.61]
8	—	—	0.63 [0.57, 0.68]	0.32 [0.24, 0.39]
9	0.55 [0.46, 0.61]	—	0.70 [0.65, 0.74]	—
10	0.52 [0.44, 0.59]	—	0.63 [0.57, 0.67]	—
11	0.39 [0.35, 0.42]	—	0.34 [0.26, 0.40]	0.74 [0.70, 0.77]
12	0.31 [0.25, 0.37]	0.52 [0.47, 0.56]	—	0.40 [0.29, 0.42]

In Table 3, Spearman’s rank correlation coefficient demonstrates that in most scenarios, environment semantics indicators show at least a “weak” correlation (0.1–0.39) to the SSR score. In most scenarios with high-intensity semantics, the correlation index is above 0.4 and can be considered “moderate” (0.4–0.69). If the semantics have high intensity over a long time, tables show “strong” (0.7–0.89) correlation (Schober et al., 2018). The table indicates that different semantics show at least moderate impacts on the SSR score; that is, the SSR score can reflect the changes in multiple semantics changes accordingly. If we connect to experiment I, we find that the results are consistent in the scope of responding to the situation changes correctly. It means our framework shows the generality ability when adding or removing semantic indicators.

Kendall’s rank correlation coefficient (Chok, 2010) is robust to outliers. Similarly, most results in Table 4 align the monotonic relationship (at least moderate positive correlation) shown in Table 3, which is expected. However, scenario 2 shows a “weak positive correlation” with noise. When we check Figure 5a, the weak correlation is reasonable. We did not use artificial noise in that scenario. Hence, the noise score is mainly affected by the robot’s movement, which stays at the bottom of the graph. It does not impact the changes in the SSR score much as we expected. A similar situation occurs in scenario 8 and results in a weak correlation. In scenario 11, there is no environment semantics showing a dominant impact on the SSR score from Figure 6b. Hence, the correlation coefficients indicate weak or moderate correlations only.

Both Spearman’s rank correlation coefficient and Kendall’s rank correlation coefficient demonstrate that our framework reveals the situation changes and can adapt the complex scenarios with multiple semantics. These environment semantics show considerable impacts on the SSR score that enable SSR as a trustworthy representative for warning situation changes.

## 7 Discussion and future work

Multi-robot deployments are expected in the future. SA is one of the prerequisites of prediction, planning, and decision-making. Our framework provides a way to obtain real-time SA that is intuitive and explainable to humans and easily usable for robots. From the scope of the experiments, the sensitivity analysis of experiment I and the Spearman’s and Kendal’s rank coefficient of experiment II, along with the analysis of the figures, reveal that our framework is sensitive enough for individual semantics situations and responds correctly in semantic-rich situations.

Compared with deep learning methods, our framework is designed to apply expert knowledge instead of data-driven training, which avoids the issue of lacking datasets. It can be potentially explainable to a human, contrary to black box models. It enables humans to understand what exactly happens in the framework and makes it more intuitive for experts tuning the framework. Human operators and robots can obtain shared SA not only from the SSR score but also from the changes in individual semantics indicators, which enables them to identify the exact situation onsite. This is crucial for real-world deployments in safety-critical applications. In addition, context and semantics can be infinite. Robots may not be able to understand all situations with a complex combination of semantics. This metric can be used to make robots aware of whether they are in a semantic-rich situation beyond their capability to understand and whether they need help from human intelligence. Hence, both the semantics indicators and the aggregated metric SSR can be used in a prediction, planning, and decision-making framework, especially from HRTs.

Regarding the flexibility and generality of our proposed approach, experiment II indicates that the framework is flexible enough to shift and comprises multiple environment semantics. It will remain robust, easily explainable, and intuitive if more environment semantics are added or removed. Depending on the applications and mission, experts can directly adjust the parameters to generalize the framework into a more reasonable representation of the given scenario. For instance, experts can highlight the weights of those semantics that are important to the goal of the mission, which makes the framework more sensitive and responsive to these semantics. The framework is not restricted to UGV deployments and can be adopted from different robotics platforms and required sensors, such as UAVs or heterogeneous multi-robot teams.

Moreover, we apply the framework in a mock-up experiment in a SAR task of a disaster-response mission (Ruan et al., 2025) context. We explored VA HRT patterns when high-level semantics are involved. Specifically, our experiments indicate that the effectiveness of the proposed framework and displaying the proposed high-level semantic indicators can help humans: decreasing reaction time when switching the level of autonomy (LoA), reducing cognitive workload, and increasing trust in their SA.

We have some limitations as well. The vision system constrains the implementation of the framework. Considering the training process, it is possible to fuse personal belonging detection and hazmat detection to simplify the deployment process, or we can adapt other state-of-the-art perception algorithms to improve the accuracy and real-time performance. However, our framework will scale nicely to continue being useful as the semantics capabilities of AI and computer vision continue to grow more powerful over time.

## 8 Conclusion

In this paper, we proposed a semantics-based SA framework to represent and quantify the variety of semantic information and the overall information richness via the concepts of environment semantic indicators and the aggregated SSR metric. We also provided an example implementation to process high-level environment semantic indicators that quantify the corresponding specific scope of the environment. Semantic perception capabilities of AI are still in an early stage of development. This is why we have chosen some relatively simple and robust examples in the experiments. However, the experiments demonstrate that our framework is capable of obtaining SA and indicate its extensibility to semantic-rich environments and has the potential to involve multiple environment semantics. The modularized design increases the flexibility, and it should adapt nicely as these AI capabilities grow.

## Data Availability

The raw data supporting the conclusions of this article will be made available by the authors, without undue reservation.
